# Vocal Creativity in Elephant Sound Production

**DOI:** 10.3390/biology10080750

**Published:** 2021-08-05

**Authors:** Angela S. Stoeger, Anton Baotic, Gunnar Heilmann

**Affiliations:** 1 Mammal Communication Lab, Department of Behavioral and Cognitive Biology, University of Vienna, 1030 Vienna, Austria; anton.baotic@univie.ac.at; 2 Gfai Tech GmbH, 12489 Berlin, Germany; mail@gunnar-heilmann.de

**Keywords:** African elephants, vocal learning, sound production, idiosyncratic sounds, vocal communication

## Abstract

**Simple Summary:**

Elephants are known for their complex vocalization system and for being able to imitate sounds. Here, we show that African elephants apply unusual and individualistic sound production mechanisms to generate idiosyncratic sounds. These sounds are produced by manipulating non-phonatory structures, e.g., applying an ingressive airflow at the trunk tip to emit extraordinarily high-frequency sounds or repeatedly contract superficial muscles at the trunk base to generate lower-frequency pulsated sounds. Intriguingly, each individual establishes its own distinctive sound-producing strategy (e.g., contracting different muscle bundles). The production of these sounds on cue is encouraged via positive reinforcement training. This suggests that social feedback and reinforcement can facilitate vocal creativity and learning behavior in elephants. Social interactions and positive feedback are also crucial for early speech learning in human infants. Increasing knowledge on sound production plasticity in elephants—long-living, highly social mammals—is crucial in the effort to better understand their communicative and vocal learning ability and its function in wild elephant populations.

**Abstract:**

How do elephants achieve their enormous vocal flexibility when communicating, imitating or creating idiosyncratic sounds? The mechanisms that underpin this trait combine motoric abilities with vocal learning processes. We demonstrate the unusual production techniques used by five African savanna elephants to create idiosyncratic sounds, which they learn to produce on cue by positive reinforcement training. The elephants generate these sounds by applying nasal tissue vibration via an ingressive airflow at the trunk tip, or by contracting defined superficial muscles at the trunk base. While the production mechanisms of the individuals performing the same sound categories are similar, they do vary in fine-tuning, revealing that each individual has its own specific sound-producing strategy. This plasticity reflects the creative and cognitive abilities associated with ‘vocal’ learning processes. The fact that these sounds were reinforced and cue-stimulated suggests that social feedback and positive reinforcement can facilitate vocal creativity and vocal learning behavior in elephants. Revealing the mechanism and the capacity for vocal learning and sound creativity is fundamental to understanding the eloquence within the elephants’ communication system. This also helps to understand the evolution of human language and of open-ended vocal systems, which build upon similar cognitive processes.

## 1. Introduction

Vocal innovation and creativity is a form of vocal learning and a core prerequisite for a flexible and open communication system. In human language, a mechanism to create new sounds, words or phrases is equally as crucial as being able to imitate sounds or lexical items outside the innate repertoire [1]. Elephants belong to the versatile but limited group of non-human mammalian species capable of complex vocal learning [2,3].

Case studies revealed vocal mimicry in two African savanna (*Loxodonta africana*) [4] and one Asian elephant (*Elephas maximus*) named Koshik, who imitated human words [5]. The faculty of vocal learning makes the elephants’ vocal system special among terrestrial non-human mammals.

Elephants produce a wide variety of sounds, both in their communication with one another as well as in the invention and imitation of sounds. Elephants use around 8–10 broad structural call categories [6,7,8,9,10,11,12]. This is not a particularly large repertoire among mammals, but elephants possess an enormous vocal plasticity, with grading between call types, call type combinations and a sophisticated within-call-type flexibility [12,13,14,15,16]. This requires control over different vocal systems, e.g., of the larynx and supra-laryngeal nasal and oral vocal tract structures in the low-frequency rumbles [10,13,16]. In trumpets, the mode of production remains speculative, although, theoretically, trumpets are incongruently high in frequency for laryngeal sound production [10,12,17]. Asian elephants have been shown to produce species-specific high-pitched squeaks, reaching a mean fundamental frequency of up to 2 kHz, by forcing air through the tensed lips, inducing self-sustained lip vibration (lip buzzing; see [17]).

Idiosyncratic sounds have been observed in captive and wild African savanna elephants [12]. These include highly variable trunk-squelching sounds, where the trunk is wriggled, wreathed or scrunched up while forcing air through it. Poole describes the squelching as giving the impression of having a ‘genuine itch’ in the trunk, but sometimes this sound itself seems to be intended [12]. Croaking is another unusual sound shown by several individuals in various facilities and in the wild; it is an orally emitted, pulsatile and harmonic sound and is often associated with sucking water or odors into the mouth [9,12]. Although the croaks of the different individuals have never been compared, their acoustic features seem similar based on available spectrograms and sound examples.

The use of non-phonatory structures to produce a variety of sounds is otherwise mainly known from aquatic mammals, i.e., pinnipeds and odontocetes, enabled by adaptations of oral and nasal structures due to an aquatic lifestyle [18,19]. Walruses (*Odobenus rosmarus*) are highly vocal, with a range of anatomical specializations that provide plasticity to their sounds. A study using contingency learning found that reinforced variability induced novelty and creativity in sounds and sound production mechanisms [20]. Besides the larynx, walruses use their pharyngeal pouches, teeth, the nose and mouth, the lips and their highly mobile tongue to produce natural and invented sounds [20].

Parrots, a long-living and social species known for their versatile ability to mimic human speech, must also be creative motorically and cognitively to utter speech sounds, considering the morphological differences between the human and avian vocal tracts. The most obvious difference is having no teeth and a beak instead of lips [21], but there are also considerable differences regarding the lungs, bronchi, the trachea or the nasal cavity [22]. Alex, an African Grey parrot, for example, has been shown involving the esophagus [23] and special tongue movements [22] to produce human speech sounds.

The pivotal question in vocal learning species that needs to be addressed is how animal vocal abilities in general connect to their cognitive and motoric capacities. In elephants, compared to species such as walruses or parrots, we have little understanding of the processing of sound-producing structures and their expressivity and no understanding of the underlying cognitive mechanisms.

Here, we describe in detail the production techniques that African savanna elephants use to generate idiosyncratic sounds trained to be produced on verbal cue via positive reinforcement. This approach reveals a remarkable individualistic variation in the production of similar vocalizations. These examples highlight the behavioral plasticity in the vocal domain, reflecting the creative and cognitive abilities associated with vocal learning processes.

## 2. Materials and Methods

### 2.1. Study Subjects

The study subjects were 5 adult African savanna elephants: Jabu (male, died July 2021) and Morula (female) from Living with Elephants in Botswana; Sawu, Mogli and Drumbo (all females) from the Dresden Zoo. All elephants were wild born. Jabu and Morula exhibited their natural behavior (e.g., foraging, mudbathing) in the bush at the Moremi Wildlife Reserve, monitored by their handler Sandi and Douglas Groves, who had direct contact with the elephants. Jabu and Morula were trained with gestural and verbal cues via positive reinforcement. Following the relevant cue, the elephants were trained to remain vocalizing until released with a release cue (‘alright’). Jabu has been with the Groves since he was a calf (when training started); Morula joined when she was 17. They were habituated to human presence. The training was not conducted in a systematic way to shape the vocalizations or production mechanisms, but rather to get them under operant control to present the sounds to visitors for educational purposes. At Dresden Zoo, the elephants are managed in a protected contact system, in which the handlers and the elephants are separated by a barrier, and are exposed to a standardized target and clicker training. Following the specific verbal cue, the elephants in Dresden are supposed to vocalize once. Again, no specific intentional guidance was applied to shape the sound; the aim here is to increase the variability of trained behavior during daily training routines. At both facilities, food functions as the primary reinforcer. In Botswana, verbal praise and patting is used as a secondary reinforcer. In Dresden, the clicker functions as a secondary reinforcer. The keepers also verbally praise elephants regularly during training sessions (another secondary reinforcer).

### 2.2. Data Collection

Idiosyncratic sounds: Data collection was conducted for one week in Botswana, and for several days at Dresden Zoo, in 2019 and 2020. Acoustic recordings were conducted using a Neumann KM183 microphone connected to a Sound Devices 633 (frequency response of the system: 10 Hz–40 kHz) at 48 kHz sampling rate and 16-bit; for video recordings, we used a Sony FD53 camcorder. For the acoustic visualization experiments, we used two different arrays; at Dresden Zoo, we used the 48-acoustic-channel Star array and the 96-acoustic-channel Mikado array. In Botswana, we used only the Mikado. Both systems measure and analyze via a delay-and-sum beamforming algorithm. The Star array has a span width of 3.4 m with 48 microphone channels (Sennheiser Electric-Capsules with MicBus microphone connectors: dynamic range 35…130 dB and 10 Hz…20 kHz), and a Baumer VLG-22C camera to provide reference images for acoustic measurement tasks. Trigger signals from the video camera enable synchronization of video images and acoustic data. The acoustic and video data were recorded using a mcdRec data recorder (http://www.gfaitech.de, accessed on 5 June 2021) at a sampling rate of 48 kHz. During recordings, the microphone array was positioned approximately 6–8 m from the elephants. Single recording sessions with this system varied between 30 and 180 s. A pre-recording trigger was set (depending on the lengths of the recordings from 30 to 90 s) so that the record button could be started once the elephant(s) had started to vocalize.

The Mikado is a new handheld system with 96 digital MEMS microphones over a 35 cm diameter surface. On-board data acquisition is provided via the DMC402L data recorder. The recording range with this system was 1 to 6 m, enabling close-up data for high-resolution sound visualization (e.g., to demonstrate biphonation).

Data collection of the general vocal repertoire used for comparison was conducted between 2003 and 2018 using an AKG 480 B CK 62 connected to a DA-P1 DAT recorder (frequency response of the system: 100 Hz:−0.2 dB, 20 Hz:−0.26 dB, 15 Hz:−0.26 dB, 12 Hz:−0.3 dB, 8 Hz:−0.32 dB, and 4 Hz:−0.45 dB). From 2011 on, we used a Neumann KM183 microphone connected to a Sound Devices 722 and the 633. Recordings were conducted at different sites including zoos, an elephant orphanage, sanctuaries and free-ranging elephants at the Addo Elephant National Park, South Africa. These recordings yielded 7419 annotated calls (Table S1). Each vocalization was visually and aurally inspected by the authors and processed using a spectrogram. Acoustic data annotation was performed using a customized annotation tool from S_Tools STx (Acoustic Institute, Austrian Academy of Science, Vienna, Austria) [24]. The start and end cues of each vocalization were tagged and the corresponding annotations were added, such as the call type; ID of the vocalizing elephants; family group and population; the age group or specific age if known; sex; broad behavioral context as well as more detailed behavioral categories; mouth posture, head, tail and ear posture; and temporal gland secretion. We also annotated overlapping calls, call combinations and choruses. The annotations were stored in XML format. In 2011 and 2012, we used the 48-acoustic-channel Star array to visualize sound emission of vocalizations at Adventures with Elephants, Bela Bela, South Africa.

### 2.3. Data Analysis

Acoustic analyses: The fundamental frequency (F0) parameter of the periodic sounds was measured using a customized semi-automatic analysis tool in Matlab. The tool takes the segmented sounds as input and computes a Fourier spectrogram. Frequency contours are then traced within the spectrogram. From these contours, a number of features are extracted automatically. We used minimum, maximum, start, mid and end and mean frequency and call duration. We also measured the peak frequency from the spectrum from both the periodic and aperiodic sounds. Of the acoustic parameters, mean values and standard deviations are reported.

Video analysis: We analyzed HD videos frame by frame observing body, articulatory and muscle movements using Solomon Coder Software Version beta 15.11.19 [25].

Sound radiation: The data were analyzed using NoiseImage 12 (http://www.gfaitech.de, accessed on 5 June 2021). The initial data, which were originally saved as channel files (*.chl), were reconverted into 2D acoustic video files (25 f/s) that could then be analyzed frame by frame. The basic principle relies on accurately calculating the specific runtime delays of acoustic sound emissions radiating from several sources to the individual microphones of the array. An acoustic map of the local sound pressure distribution at a given distance is calculated by a delay-and-sum beamforming algorithm using the acoustic data of all simultaneously recorded microphone channels. The sound pressure level (SPL) is displayed by color coding. The automatic overlay of optical image and acoustic map allows the locations of dominating sound sources to be identified. NoiseImage enables adjusting the focus post-recording to locate the sound source in still images even from moving objects. Frequency ranges of specific interest can be manually selected from the spectrogram and then only these are displayed on the acoustic map in the corresponding 2D acoustic photo. N_visualization_ gives the number of successful sound visualizations per individual and call type out of the total number of sound visualization trials (this number is given in parentheses). There were several possible reasons for unsuccessful visualizations, including the elephant moving its head or body, the body part in question was out of focus (e.g., moving the trunk out of focus), windy conditions, loud background noise or too much backlight. However, due to the prerecording trigger, unsuccessful trials were often not saved and could be reduced to a minimum.

For graphical comparison of the periodic sounds, we calculated mean F0 and used call duration as well as sound production parameters (sound radiation, mouth posture and respiration phase). The 3D scatter plot was computed using the Plotly package in R [26]. For the scatter plot, we used 348 rumbles, 143 trumpets, 109 roars and 25 barks. Of these data, 4 rumbles were from Sawu, 9 from Drumbo and 4 from Mogli, as well as 2 trumpets from Drumbo and one from Mogli. Twelve rumbles from Jabu and 6 rumbles from Morula (both from Botswana) are also in the data set. In addition, we used 19 croaks (recorded at Vienna Zoo) and 171 HFSs.

## 3. Results

Elephant calls are best classified along multiple dimensions of acoustic parameters, i.e., overall periodicity (periodic vocalizations with measurable F0, versus aperiodic calls), call duration and sound production mechanisms. We found periodic as well as aperiodic idiosyncratic sounds.

### 3.1. Periodic Idiosyncratic Sounds

**Figure 1:**
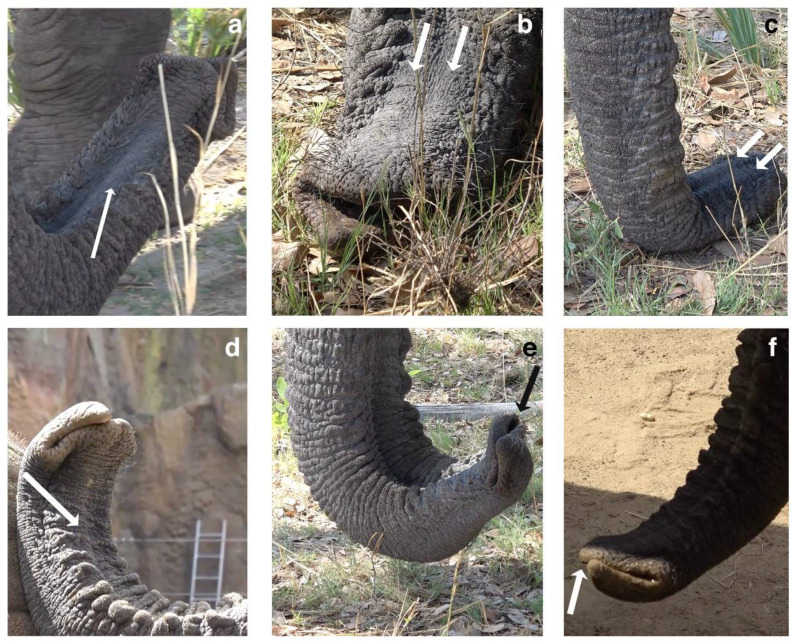
Observations during high-frequency sound (HFS) production. (**a**) shows Jabu stiffening only his left nasal tube during HFS production. (**b**) Jabu’s trunk is resting on the ground (not vocalizing), both nasal tubes are quite relaxed. (**c**) shows Morula’s trunk (not vocalizing), both of her nasal tubes are stiffened and clearly visible. (**d**) Sawu during HFS production, stiffening and closing off her right nasal tube. (**e**) Jabu during HFS production: his left nasal tube is closed, the opening of the right one is visible and marked with the black arrow. (**f**) shows how Sawu is actually crossing the fingers of the trunk during HFS production.

We documented high-pitched idiosyncratic sounds, which we termed high-frequency sound (HFS), by Jabu, Morula and Sawu. Sound emission in all three individuals (N_Morula_ = 14(25), N_Jabu_ = 19(42), N_Sawu_ = 4(8)) was detected at the trunk tip. All three elephants pressed the trunk tip together, closing off one nostril while sucking in air through the other. Although the sound quality was similar, the acoustic structure varied. Sawu’s HFSs, with a mean F0 of 1860 ± 285 Hz (N = 37), are considerably higher and shorter in duration (0.58 ± 0.17 s, N = 37) than Jabu’s (445.69 ± 59.87 Hz, 2.43 ± 0.93 s, N = 92) and Morula’s (391.80 ± 242.74 Hz, 1.17 ± 0.59 s, N = 50) (Table 1). Jabu’s peak frequency is the second harmonic, and not the fundamental as in Sawu and Morula (Table 1). Jabu tilts the tip of his trunk to the left while stiffening and tensing the left nasal tube at the end of the trunk (Video S1, Figure 1a). A stiffened/tensed nasal tube is visible (as a duct) at the area near to the trunk tip (Figure 1a–d). Morula turns the trunk tip upwards, also putting more tension on the left nasal tube (both initially close off the left nostril, Figure 1e). Sawu slightly tilts the trunk tip to the right, tensing her right nasal tube (closing off her right nostril) (Video S1, Figure 1d); see Table 2.

**Figure 2:**
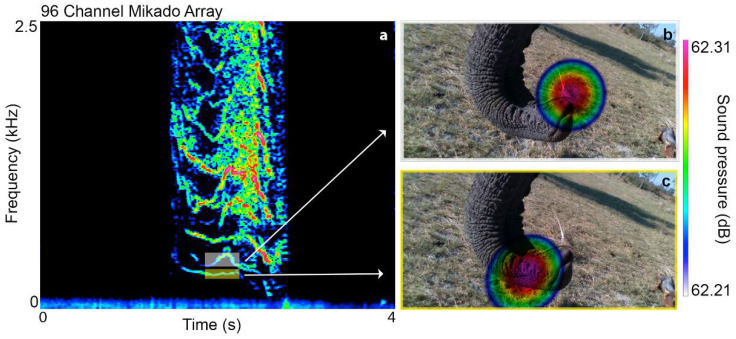
Biphonation in a high-frequency sound. Spectrogram (**a**) and sound visualizations (**b**,**c**) of a biphonation event in a HFS produced by Jabu, revealing that the two independent frequencies (yellow and white rectangle) are emitted via different nostrils.

The HFSs of all individuals feature nonlinear phenomena (NLP, Table 1) typical of self-oscillating systems when driven to the limit or where multiple oscillators interact [27]. The most prominent NLP was biphonation (67%), characterized by the incidence of two independent frequencies. Biphonation in HFSs occurs—though perhaps not exclusively—when occlusion of the closed nostril becomes leaky and air is being sucked in through both nostrils simultaneously, resulting in two independent sound sources (Figure 2).

**Figure 3:**
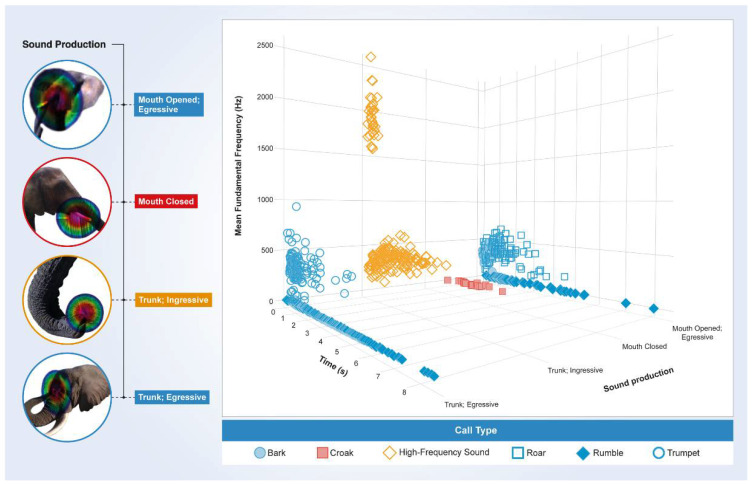
Comparison of periodic vocalizations. Three-dimensional scatter plot (duration, F0 and sound production) showing 625 vocalizations with measurable F0 of adult, adolescent and calf African savanna elephants (blue icons), in comparison with the idiosyncratic croak and the novel HFS. Morula’s and Jabu’s HFSs are considerably lower (all HFSs below 1000 Hz) than Sawu’s (all HFSs above 1500 Hz). These sounds, particularly the HFSs from Sawu, are special because the highest-pitched calls documented in adult African elephants are trumpets and roars reaching mean fundamental frequencies of around 500 Hz maximum. Supplementary Figure S1 gives an interactive version of this scatter plot to view the data from different angles and perspectives.

The 3D graphical comparison of periodic vocalizations with duration, mean F0 and production mechanisms for each represented call (collapsed into one dimension) reveals that the HFSs stand out (particularly in frequency and sound production mechanism) against a selection of 625 African elephant adult, adolescent and calf vocalizations (Figure 3).

### 3.2. Aperiodic Idiosyncratic Sounds

**Figure 4:**
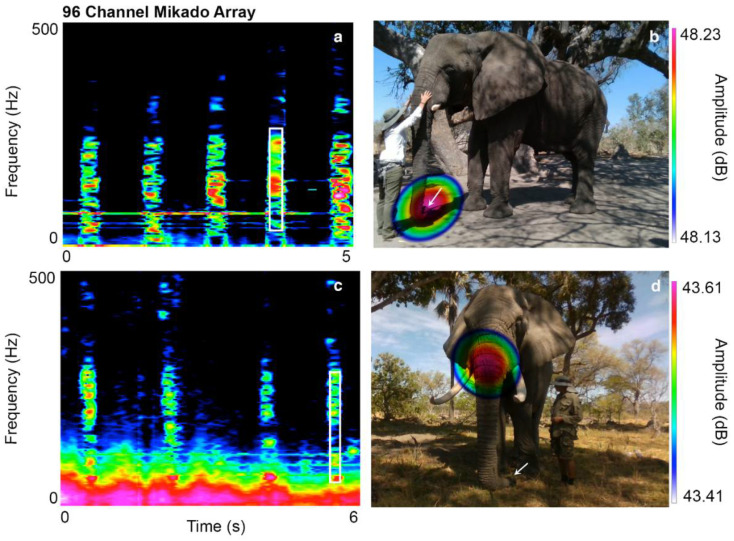
Structure and sound radiation of throb sounds. Spectrograms and sound visualizations of Morula’s (**a**,**b**) and Jabu’s (**c**,**d**) throb sounds. In Morula, sound emission was detected at the curled trunk tip; in Jabu, sound emission appeared below the trunk base, and the trunk tip rests on the ground.

Two unusual aperiodic sounds were documented. The throb sound is a pulsed and aperiodic vocalization with emphasized frequency regions, documented in Jabu (duration = 0.47 ± 0.054 s, peak frequency = 181 ± 16.74 Hz, N = 30) and Morula (duration = 0.51 ± 0.086 s, peak frequency = 128.56 ± 14.34 Hz, N = 30). These vocalizations are produced via contractions of superficial trunk muscles at the upper nasal vocal tract. Although the throb sounds are similar in structure, Morula and Jabu use different mechanisms (Table 2). Morula contracts the longitudinal muscle bundles of the *maxillo labialis* (that originally function to lift the trunk) directly below the forehead covering the nasal cavity [28,29] (Video S2). Sound emission was detected at the curled trunk tip (analogous to making a fist, N_visualization_ = 9(15), Figure 4a,b). Jabu contracts the paired *musculus nasalis* [28,29], a helical muscle that helps twist the upper trunk [26,27] (Video S2). Sound radiation occurs directly below the trunk base, and the trunk tip relaxes on the ground (N_visualization_ = 10(15), Figure 4c,d).

**Figure 5:**
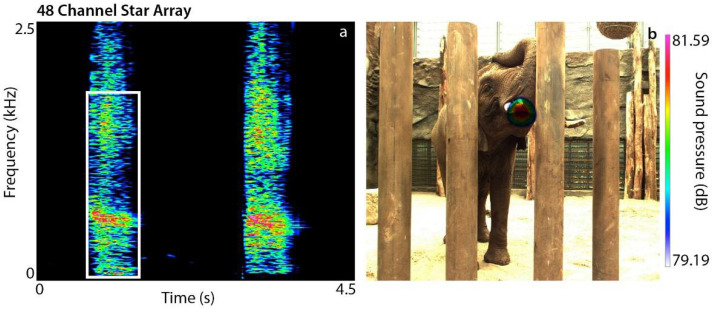
Structure and sound radiation of oral bursts. (**a**) Spectrogram and (**b**) sound visualization of an oral burst produced by Drumbo. The white square indicates the selected frequency range for sound visualization.

When producing the oral burst (duration = 0.72 ± 0.31 s, peak frequency = 461.54 ± 61.73 Hz, N = 37, N_visualization_: Drumbo = 7(7), Mogli = 4(5), Sawu = 3(5)) (Figure 5), air is blocked by posteriorly obstructing the oral chamber and is then suddenly released, causing an abrupt burst of sound and vibrations of the soft palate (Video S3, Table 2). The sound structure is very similar among the three individuals (Table 1).

## 4. Discussion

The HFS, the throb sound and the oral burst are sound categories generated by manipulating non-phonatory structures. The production mechanisms of the individuals performing the HFS and the throbbing sounds are similar but vary in fine-tuning, indicating that individuals established specific strategies. All vocalizations are reliably emitted in response to verbal cues given by their handlers, which reveals profound volitional control over these production techniques [30].

Operant reinforcement presupposes processes that generate novel behavior in advance of selection. Accordingly, a sound must occur before it can be reinforced [31]. Sawu from Dresden was co-housed with a female Asian elephant until 2008. She might have been imitating her co-inhabitants’ high-pitched squeaks (Sawu is significantly higher in pitch than Jabu and Morula), yet establishing a different production mechanism. The HFS is, to our knowledge, the first reported ingressive elephant vocalization (considering all elephant species). Based on the acoustic structure with considerable sound energy even in the upper harmonics, we suggest nasal tissue vibration during inhalation as the sound-producing mechanism (whereas most Asian elephants use lip buzzing during exhalation). Another possible mechanism to generate high-frequency sounds would be whistling, as in the wapiti [32], or in the pursed lips of walruses [33] and humans [34]. Whistling, however, produces tonal, almost sinusoidal sounds, with most of the energy located in the fundamental frequency [35]. In HFSs, the upper harmonics also possess considerable energy; in fact, Jabu’s peak frequency is the second harmonic, not the fundamental frequency. Jabu was raised by his handlers since he was orphaned at 2 years of age. When relaxed, he often played with his trunk and the resulting sounds (Sandi Groves, personal communication). These sounds were then reinforced to be emitted on cue and perhaps modified and shaped during training. Nonetheless, the sound and the production mechanism have not been intentionally modified by the trainers (personal communication). Morula joined Jabu when she was 17 and started producing HFSs that were then also reinforced in training. Details of the learning processes are not known.

The throb sound was first produced by Jabu. Later, Morula started as well (Sandi Groves, personal communication), but used different muscles to generate the sound. Moreover, Morula’s sound emission occurs at the trunk tip, not at the base of the trunk as in Jabu. Our theory is that Morula’s nasal vocal tract is activated and the throb sound travels down the nostrils. Jabu, instead, might close off the nasal vocal tract at a different location in order to increase air pressure for sound production at the source, since his trunk tip remains relaxed during throbbing. Muscle movements in elephants are often visible at the forehead and at the base of the trunk while moving the trunk, while manipulating objects, sucking or inhaling odors (this muscle movement is referred to as nasal throbbing [36]). The throb sound in its current specificity, documented in Jabu and Morula, might have originated from such muscle movements. Nonetheless, the elephants use only defined muscles bundles to generate the specific sounds. To our knowledge, repeatedly and selectively contracting the *musculus nasalis* or *musculs lateralis* for sound production has not been described as such before. In nasal rumbles, a passive fluttering of the forehead is visible, but this is caused by air originating from the lungs, passing through the larynx (the sound source) and the nasal vocal tract. In the throb sound, these muscles, in fact, generate the sound.

The production of these sounds also differs from trumpets and snorts. Trumpets have been suggested to be produced via vibrations of the margin of rigid cartilaginous plates lateral of the nasal cavity [37], caused by forceful exhalation of air through the nasal vocal tract (Figure S1i,j) [10,12]. The snort also seems to be produced by air blown through nasal cavities, but with less power and force than in a trumpet (Figure S1k,l). Elephants trained to trumpet on cue tend to snort if not executing the request properly, and then start trumpeting when asked by the trainer to repeat the sound with more force in order to get the reward (Video S4) [30]. This indicates a similar sound production mechanism, but with varying effort. The documented throb sounds also differ considerably from trunk squelching in their mode of production. In squelching, the entire trunk moves in a concertina hose manner [12] (Video S5), whereas during throbbing, the trunks remain static (except for the locally confined contractions of the defined muscles).

In the case of the oral burst from the Dresden elephants, the handler reinforced a specific sound he heard while the elephants were swallowing, which then led to the development of the current oral burst (Ronny Moche, personal communication). We could only observe in detail (filming into the open mouth) the production in one individual, but the observable mechanism (oral emission) as well the acoustic structure are very similar in all three individuals. Otherwise, noisy and mixed roars are the most common aperiodic sounds emitted orally (Figure S1e,f). However, in these calls, the mouth is wide open and the most likely production mechanism is passive vocal fold vibration (as in rumbles). The air, however, is passed through with greater force than in an orally emitted rumble (Figure S1c,d), causing irregular vocal fold vibrations that yield an overall aperiodic call structure.

Since none of the elephants were trained for the purpose of this study, we can document only the ‘final’ sounds and production mechanisms, but not the developmental processes involved. Initially, the HFS or the throbbing sound could have been a modification of an existing behavior or sound, an invention and/or an imitation. The ‘final’ sounds and their production mechanisms that we observed might, nonetheless, be a result of a specific shaping by the trainer (even if unintentionally) and/or represent invention processes that the elephants used to fulfill the training requirements. Importantly, training, regardless of the way it occurs, involves learning by reinforcement.

Motivation and social circuits of the brain are intimately connected, predisposing social individuals to attach reward value to social partners [38,39,40]. The role of social influences, feedback and reinforcement on vocal learning in non-human animals is still relatively poorly understood [41,42,43] even though social interactions and positive feedback are crucial for early speech learning in human infants [44,45]. Socially guided vocal learning is thought to require additional connections between the social motivation system and the vocal learning system [46]. The zebra finch, the most common model of human speech development, was long thought to learn only via imitation. Carouso-Peck and Goldstein [47], however, recently showed that song learning in young males is positively affected by non-vocal, visual feedback from females. In parrots, different types of vocal learning behavior require a different feedback or input [48]. Pet parrots may mimic random words and environmental noises without clear instruction, but parrots acquire communication skills most effectively when teaching is ‘functional and referential, and socially rich’ [48,49]. Social interaction with trainers engages the animals directly; they get a contextual explanation and consequences for actions [49].

In non-human mammals, socially guided vocal learning was reported in killer whales (*Orcinus orca*) that were cross-socialized with bottlenose dolphins [50]. In marmosets (*Callathrix jacchus*), experiments with twins revealed that infants who received more contingent feedback had a faster rate of vocal development, producing mature-sounding contact calls earlier than the other twin [51]. Calimero, a male African elephant that was cross-socialized and raised among Asian females, imitated their high-frequency and repetitive sounds [4]. Here, similarly, social bonding along with social feedback by the Asian elephants might have been the determining factors for imitation to occur. Elephants, like humans, are terrestrial, long-living and highly social mammals interacting in a complex fission–fusion society [52,53,54]. Accordingly, social environment and social interactions play a crucial role in communication, and vocal learning in elephants might be driven by social motivation. During training, the social partners interacting with the elephants are the handlers, and not conspecifics. At the same time, elephants and handlers are known to establish social bonds with positive effects, including operational and affective benefits on both sides [55,56,57].

In this paper, we specifically show variation in sound production of similar sounds by individuals (that are trained to produce them on cue) and suggest that social feedback and reinforcement facilitate elephant vocal learning behavior in general. This opens up the opportunity to conduct controlled and guided experiments (also including contingency learning) to examine how elephants learn to invent or imitate sounds. This would help to reveal the underlying mechanisms of their vocal plasticity.

It remains to be explored how this relates to the behavior of wild elephant populations. Nonetheless, determining these skills in trained individuals is a valuable and necessary step forward to finally explore and reveal the relevance and functional adaptation of the vocal learning ability within the elephants’ communication system. It is fundamental to understand the observed expressivity and variability of their vocalizations in the wild, and this will help to further improve our understanding of the evolution of the vocal learning trait that is so important for human speech and language.

## Figures and Tables

**Table 1:** Acoustic features of the idiosyncratic sounds. Fundamental frequency parameter or mean peak frequency, duration and % non-linear phenomena (in HFS) of each individual, respectively.

**High-Frequency Sound**			
**Parameter**	**Jabu (N = 92)**	**Morula (N = 50)**	**Sawu (N = 37)**
F0 start ± SD (Hz)	554.66 ± 156.02	541.48 ± 299.22	2141.31 ± 385.78
F0 mid ± SD (Hz)	454.20 ± 71.669	404.08 ± 279.84	1854.62 ± 331.98
F0 end ± SD (Hz)	350.20 ± 100.82	313.72 ± 202.56	1672.32 ± 398.29
F0 minimum ± SD (Hz)	345.90 ± 99.43	299.20 ± 204.13	1696.34 ± 269.33
F0 maximum ± SD (Hz)	578.70 ±140.89	563.16 ± 332.78	2161.57 ± 392.13
F0 mean ± SD (Hz)	444.69 ± 59.87	391.80 ± 242.74	1859.90 ± 285.14
Peak frequency ± SD (Hz)	1182.03 ± 223.53	406.08 ± 253.35	2011.16 ± 330.32
Duration ± SD (s)	2.43 ± 0.93	1.17 ± 0.59	0.58 ± 0.17
% biphonation	75.6	63.3	43.2
% subharmonics	47.2	12.2	43.6
% frequency jumps	29.3	8.2	16.2
% chaos	3.3	----	13.5
**Throb Sound**			
	**Jabu (N = 92)**	**Morula (N = 50)**	
Peak frequency ± SD (Hz)	82.79 ± 50.38	128.56 ± 14.34	
Duration ± SD (s)	0.47 ± 0.054	0.51 ± 0.086	
**Oral Burst**			
	**Drumbo (N = 15)**	**Mogli (N = 10)**	**Sawu (N = 12)**
Peak frequency ± SD (Hz)	468 ± 21.99	461 ± 23.56	445 ± 78.86
Duration ± SD (s)	1.11 ± 0.255	1.01 ± 0.192	0.58 ± 0.247

**Table 2:** Idiosyncratic sounds and variation of sound production in individuals.

Individual	Sound Type	Sound Emission	Respiratory Phase	Description
Jabu	HFS	Trunk tip	Ingressive sound	Tilts the tip of his trunk to the left, while stiffening and closing the left nasal tube.
Morula	HFS	Trunk tip	Ingressive sound	Tilts the tip of her trunk upwards, stiffening and closing the left nasal tube.
Sawu	HFS	Trunk tip	Ingressive sound	Tilts the tip of her trunk slightly to the right, stiffening and closing the right nasal tube.
Jabu	Throb sound	Trunk base	Egressive sound	Contractions of *musculus nasalis.*
Morula	Throb sound	Trunk tip	Egressive sound	Contractions of the *maxillo labialis* at the trunk base.
Mogli	Oral burst	Mouth	Egressive sound	Vibration of soft palate: air blocked by a posterior obstruction of the oral chamber, then abruptly released, causing a burst of sound.
Drumbo	Oral burst	Mouth	Egressive sound	Not known, most likely similar to Mogli.
Sawu	Oral burst	Mouth	Egressive sound	Not known, most likely similar to Mogli.

## Data Availability

All data will be available upon request.

## Supplementary Materials

The following are available online at https://www.mdpi.com/article/10.3390/biology10080750/s1, Table S1: Available data used to compare and discriminate idiosyncratic sounds from the commonly occurring vocalizations, Figure S1: 3D_scatterplot.html Interactive Map Data. Three-dimensional scatter plot (*.html) comparing of periodic natural and novel vocalizations. Figure S2: Broad call type categories in African savanna elephants. Specifically, the spectrogram and the respective sound visualization is given of a nasal rumble (Figure S2a,b), an oral rumble (Figure S2c,d), a noisy roar (Figure S2e,d), a bark (Figure S2g,h), a trumpet (Figure S2i,j) and of a snort (Figure S2k,l). Video S1: High-frequency sound produced by Jabu (Botswana) and Sawu (Dresden). Video S2: Throb sounds produced by Jabu and Morula. Video S3: Oral bursts produced by Mogli at the Dresden Zoo. Video S4: Video of Iqhwa (an 8-year-old female at Vienna Zoo, Austria) performing trumpet sounds in response to the cue “Laut”. Video S5: Video of an adult male at Pilanesberg Back Safaris producing a trunk squelch.
