# Supplementary material for: Vocal Creativity in Elephant Sound Production
Source: Biology (Basel). 2021 Aug 5;10(8):750. doi: 10.3390/biology10080750 (PMC8389636; doi:10.3390/biology10080750)
Supplement: Supplementary file 1 [file biology-10-00750-s001.zip › Supplementary material.pdf]

Supporting Information for

Vocal creativity in elephant sound production

Angela S. Stoeger, Anton Baotic, Gunnar Heilmann
Correspondence to: angela.stoeger-horwath@univie.ac.at

Table S1. Available data used to compare and discriminate idiosyncratic sounds from the commonly occurring vocalizations.
The table gives the respective dataset with facility information, the age groups and sex of the study subjects, and the number of calls annotated. Each vocalization was visually and aurally inspected by the authors and processed using a spectrogram. Start and end cues of each vocalization were tagged and the corresponding annotations were added, such as the call type, ID of the vocalizing elephants, family group and population, the age group or specific age if known, sex, broad behavioral context as well more detailed behavioral categories, mouth posture, head, tail, ear posture, temporal gland secretion were annotated.

Dataset (facility information)	Subjects	N calls annotated
Vienna Zoo 2003 and 2004	adult ♀	941
Vienna Zoo 2003/2004,<br>Daphne Sheldrick's Orphanage 2004	infant, calves until 2.5 years ♂ ♀	1491
Adventures with Elephants 2011 and 2012	Subadult ♂ ♀	440
Addo Elephant National Park 2011 and 2012	adult, juvenile, calves, infant ♂ ♀	1325 + 1031
Vienna Zoo 2013	adult, juvenile, calves, infant ♂ ♀	119
Adventures with Elephants 2014	Subadult ♂ ♀	465
Elephant Whispers 2014	adult, subadult ♂ ♀	415
Pilanesberg Back Safaris 2014	adult, juvenile, infant ♂ ♀	263
Addo Elephant Back Safaris 2015 and 2016	adult ♂	70 + 16
Vienna Zoo and Berlin Zoo 2016	adult, subadult, juvenile, calves ♂♀	182
Adventures with Elephants 2018	adults, subadults, calves ♂ ♀	661

Figure S1. 3D_scatterplot.html
Three-dimensional scatter plot (*.html) comparing of periodic vocalizations of the common repertoire and the idiosyncratic sounds croak and HFS.

Scatter plot of duration, F0 and sound production showing 625 vocalizations (barks, trumpets, roars, rumbles) of adult, adolescent, and calf African savanna elephants (blue icons), in comparison with the periodic idiosyncratic croak (red squares) and the HFSs (yellow diamonds).
Morula's and Jabu's HFSs are considerably lower (all HFSs below 1000 Hz) than Sawu's (all HFSs above 1500 Hz). To view the data from different angles and perspectives you may simply click (and hold) on the plot and move it to the position of interest. One also may hover above the logos at the top-right corner to use the function in the appearing menu bar to zoom in or out to explore call type distribution in more detail.

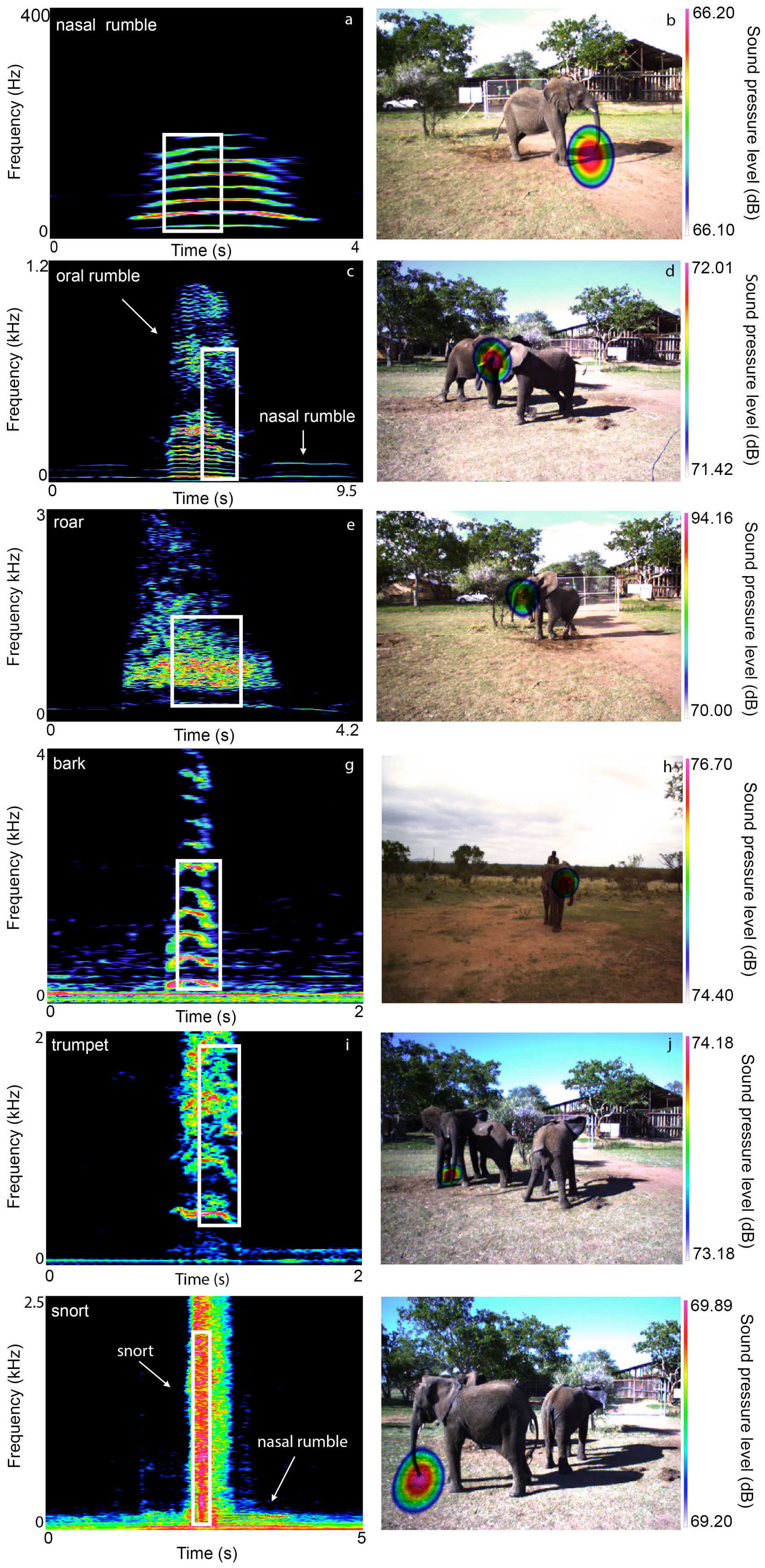

Figure S2. Spectrograms and the respective sound visualization side by side of the most common sound categories in African savanna elephant communication (the names of the call types are given in each left panel), recorded with the 48 Channel Star Array. Data were collected in 2011 at Adventures with Elephants, in Bela Bela, South Africa.

Video description

Video S1. High-Frequency Sound produced by Jabu (Botswana) and Sawu (Dresden). We suggest that HFSs are produced by nasal tissue (at the tip of the trunk) set into vibration via an ingressive airflow. In this setting it is evident that at the beginning of the sound, Jabu tilts the tip of his trunk to the left while stiffening and tensing the left nasal tube, which thus becomes visible as a duct. The right tube seems to remain relaxed, which apparently enables tissue vibration. Sawu slightly tilts the trunk tip to the right, tensing her right nasal tube (closing off her right nostril). In the video, the keeper gives the verbal command to vocalize ("squeal"), and then Sawu vocalizes. Right after the sound, Sawu is reinforced via the clicker (a conditioned reinforcer) and food. For a high-resolution video, use the following link: https://youtu.be/78DEj1SRf0M

Video S2. Throb-sounds produced by Jabu and Morula. Throb-sounds appear to be produced via contractions of superficial trunk muscles at the upper nasal vocal tract. Jabu seems to contract the paired *musculus nasalis*. Morula contracts longitudinal muscle bundles of the *maxillo labialis* directly below the forehead covering the nasal cavity. For a high-resolution video, use the following link: https://youtu.be/Ysd4wRmsllU

Video S3. Oral bursts produced by Mogli at the Dresden Zoo. In order to produce an oral burst, air is blocked by posteriorly obstructing the oral chamber and is then suddenly released, causing an abrupt burst of sound and vibrations of the soft palate. In the video, the keeper gives the verbal command to vocalize ("speak"), and then Mogli vocalizes. Right after the sound, she is reinforced via the clicker (a conditioned reinforcer), some fruits and verbal praise. For a high-resolution video, use the following link: https://youtu.be/xMESEhXFP2Q

Video S4. Video of Iqhwa (an 8-year old female at Vienna Zoo, Austria) performing trumpet sounds in response to the cue "Laut". The first sound is rather a snort (but she is verbally reinforced), in the second vocalization the trumpet sound can be perceived, the third one is best and is reinforced with the clicker and a food reward. For a high-resolution video, use the following link: https://youtu.be/xIuW8I2clu4

Video S5. Video of an adult male at Pilanesberg producing a trunk squelch. He is wriggling his trunk, and you can hear the bubbling sound, as described by Poole 2011. However, trunk squelching sounds are highly variable. For a high-resolution video, use the following link: https://youtu.be/8FSopdjK7rc

Reference:

Poole, J.H. Behavioral contexts of elephant acoustic communication. In *The Amboseli Elephants: A Long-Term Perspective on a Long-Lived Mammal*; Moss, C.J., Croze, H., Lee, P.C., Eds.; Chicago University Press: Chicago, IL, USA, 2011; pp. 125-161.
